# Improved Self-Calibration of a Multilateration System Based on Absolute Distance Measurement

**DOI:** 10.3390/s20247288

**Published:** 2020-12-18

**Authors:** Quoc Khanh Nguyen, Seungman Kim, Seong-Heum Han, Seung-Kook Ro, Seung-Woo Kim, Young-Jin Kim, Wooram Kim, Jeong Seok Oh

**Affiliations:** 1 Department of Ultra-Precision Machines and Systems, Korea Institute of Machinery and Materials, Daejeon 34103, Korea; khanh310@kimm.re.kr (Q.K.N.); kimsm@kimm.re.kr (S.K.); sh-han@kimm.re.kr (S.-H.H.); cniz@kimm.re.kr (S.-K.R.); 2 Department of Nano-Mechatronics, University of Science and Technology, Daejeon 34113, Korea; 3 Department of Mechanical Engineering, Korea Advanced Institute of Science and Technology, Daejeon 34141, Korea; swk@kaist.ac.kr (S.-W.K.); yj.kim@kaist.ac.kr (Y.-J.K.); kwr0704@kaist.ac.kr (W.K.)

**Keywords:** multilateration, self-calibration, absolute distance measurement, initial guess

## Abstract

Multilateration tracking systems (MLTSs) are used in industrial three-dimensional (3D) coordinate measuring applications. For high-precision measurement, system parameters must be calibrated properly in advance. For an MLTS using absolute distance measurement (ADM), the conventional self-calibration method significantly reduces estimation efficiency because all system parameters are estimated simultaneously using a complicated residual function. This paper presents a novel self-calibration method that optimizes ADM to reduce the number of system parameters via highly precise and separate estimations of dead paths. Therefore, the residual function to estimate the tracking station locations can be simplified. By applying a suitable mathematical procedure and solving the initial guess problem without the aid of an external device, estimation accuracy of the system parameters is significantly improved. In three self-calibration experiments, with ADM repeatability of approximately 3.4 µm, the maximum deviation of the system parameters estimated by the proposed self-calibration method was 68.6 µm, while the maximum deviation estimated by the conventional self-calibration method was 711.9 µm. Validation of 3D coordinate measurements in a 1000 mm × 1000 mm × 1000 mm volume showed good agreement between the proposed ADM-based MLTS and a commercial laser tracker, where the maximum difference based on the standard deviation was 17.7 µm. Conversely, the maximum difference was 98.8 µm using the conventional self-calibration method. These results confirmed the efficiency and feasibility of the proposed self-calibration method.

## 1. Introduction

In recent decades, the demand for accurate measurement of large structures has increased considerably. Accordingly, large-scale metrology has played an important role in industrial applications [1,2]. Since the first interferometry-based prototype was introduced by Lau et al. in the 1980s [3,4], laser tracking systems have been widely used in many engineering applications such as robot metrology, precision machine calibration, automotive and aircraft assembly, and industrial shipbuilding [1,2,5]. A single laser tracker incorporates angle sensors on two rotational axes, which carry the interferometer, thus providing a spherical coordinate measurement system. Moreover, the use of absolute distance measurement (ADM) offers the convenience of re-establishing distance data after a beam-break event, as well as dynamic measurement related to speed improvement [5,6]. However, the uncertainty associated with angular information remains the main source of error in large-volume measurement [7]. Efforts to reduce this error have led to the use of multilateration tracking systems (MLTSs), in which angle measurement is entirely removed and only multi-distance information is used for three-dimensional (3D) coordinate measurement [8]. Theoretically, an MLTS could improve measurement accuracy if the remaining uncertainty sources (e.g., system configuration and the self-calibration process) are carefully considered [9]. The conventional self-calibration method for incremental distance measurement (IDM)-based MLTSs has been extensively studied to enhance overall performance in 3D coordinate measurement [10,11,12,13]. However, to the best of our knowledge, there have been no reports of a custom-designed self-calibration method for ADM-based MLTSs, which could implement advantages of ADM to provide high-precision estimation of system parameters (i.e., dead paths and tracking station locations).

The conventional self-calibration algorithm for a multilateration system using IDM was developed and implemented by Zhuang et al. [10] and Takasuji et al. [11,12,13]. In detail, before calculation of the 3D coordinates of target points, all system parameters are simultaneously estimated by minimizing a nonlinear residual function [13]. Because all system parameters must be repeatedly calibrated at the beginning of a new measurement, estimation of those parameters can become unstable in some circumstances. Thus, the optimization process may lead to poor estimation or convergence failure [13,14]. Furthermore, the lack of an analytical calculation of the initial guess, performed to overcome the local minimum problem of nonlinear optimization process, could be a critical problem when good initial guesses of the system parameters are not obtained [15,16].

With the aim of providing a solution to the above-mentioned issues, this paper presents a novel self-calibration method for an MLTS using ADM, in which the advantages of ADM are effectively optimized to improve the efficiency of the self-calibration process and provide good initial guesses for least squares estimation. In detail, the self-calibration process is divided into two steps: estimation of dead paths and estimation of tracking station locations. Here, the tracking station is a device for tracking a target in 3D space and measuring the absolute distance to the target, and the dead path is the offset distance between the origin of ADM and the tracking station location (i.e., rotating center of tracking station). A small number of system parameters are then estimated in each step, which reduces the complexity of the original residual function. Moreover, when precise dead paths have been obtained, re-estimation is not required [5]. Notably, by using the roughly estimated elevation angle offset, the initial guess problem can be solved automatically without the aid of any external device. This enables improvement of the on-site calibration of tracking station locations. By combining the two-step self-calibration approach and automated calculation of the initial guesses, the self-calibration performance can be considerably improved. Finally, the estimated system parameters are ready for use in actual measurement applications.

The remainder of this paper is organized as follows. Section 2 provides the background of the conventional self-calibration method. Section 3 presents the detailed mathematical model of the proposed self-calibration method in two parts: dead-path estimation and on-site calibration of tracking station locations. Section 4 shows the simulation results and experimental findings.

## 2. Conventional Self-Calibration Method for 3D Coordinate Measurement

**Figure 1:**
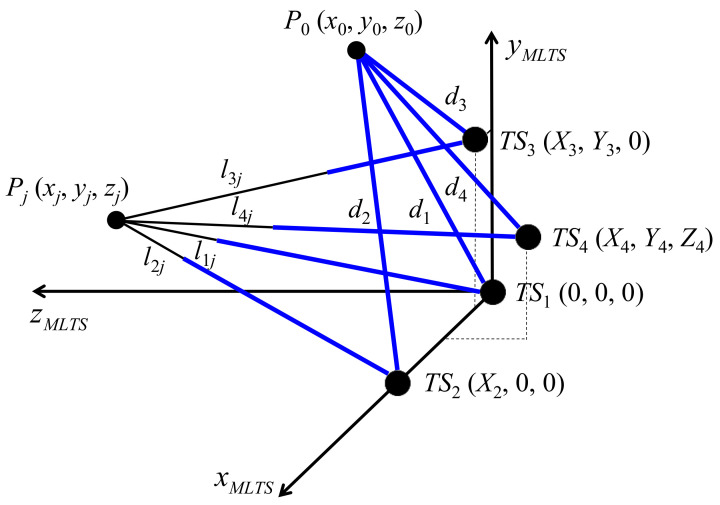
Schematic of the conventional self-calibration method for a multilateration tracking system using incremental distance measurement.

The schematic in Figure 1 depicts the conventional self-calibration method for an MLTS using IDM, in which the typical locations of four tracking stations are selected in the MLTS coordinate system as follows: *TS*_1_(0, 0, 0), *TS*_2_(*X*_2_, 0, 0), *TS*_3_(*X*_3_, *Y*_3_, 0), and *TS*_4_(*X*_4_, *Y*_4_, *Z*_4_). Theoretically, the MLTS uses at least three tracking stations. The 3D coordinates of the target point are then identified using the trilateration formula [10]. However, information concerning the locations of tracking stations (*X*_2_, *X*_3_, *Y*_3_, *X*_4_, *Y*_4_, and *Z*_4_) must be known in advance. Furthermore, only the relative distance *l_ij_* is measured in an IDM-based MLTS. Thus, the initial lengths (*d*_1_, *d*_2_, *d*_3_, and *d*_4_), which are defined as the distance from each tracking station to initial point *P*_0_ (where interferometers are reset to zero), must be known to calculate the exact distance from each tracking station to target point *P_j_*(*x_j_*, *y_j_*, *z_j_*).

In this circumstance, the concept of self-calibration is proposed, whereby inclusion of an additional tracking station provides an over-determined system, in contrast to a system that uses an external device for calibration. The initial lengths and locations of tracking stations can then be self-calibrated. The original residual function used in the conventional self-calibration method [13] is as follows:

(1) $${\;R}_{ori}\;=\overset{4}{\underset{i=1}{\sum }}\overset{n}{\underset{j=1}{\sum }}{\left\{{\left[{{(x}_{j}\;-{\;X}_{i})}^{2}{\;+\;(y}_{j}\;-{\;Y}_{i}{)}^{2}{\;+\;(z}_{j}\;-{\;Z}_{i}{)}^{2}\right]}^{1/2}-{\;(d}_{i}{\;+\;l}_{ij})\right\}}^{2}$$

where *n* is the number of measurement points for self-calibration, *X_i_*, *Y_i_*, *Z_i_* represent the location of the *i*^th^ tracking station, *x_j_*, *y_j_*, *z_j_* are coordinates of measurement point *P_j_*, *d_i_* is the initial length of the *i*^th^ tracking station, and *l_ij_* is the relative distance determined by IDM. A simpler version that helps to reduce the complexity of the residual function has been proposed to improve estimation accuracy [13]. This simplified equation is as follows:

(2) $${\;R}_{ori\_sim}\;=\;\overset{n}{\underset{j=1}{\sum }}{\left\{{\left[{{(x}_{j}\;-{\;X}_{4})}^{2}{\;+\;(y}_{j}\;-{\;Y}_{4}{)}^{2}{\;+\;(z}_{j}\;-{\;Z}_{4}{)}^{2}\right]}^{1/2}-{\;(d}_{4}{\;+\;l}_{4j})\right\}}^{2}$$

As specified in Equations (1) and (2), all system parameters (i.e., initial lengths and locations of tracking stations) are simultaneously estimated in the conventional self-calibration method.

## 3. Proposed Self-Calibration Method for an ADM-Based MLTS

**Figure 2:**
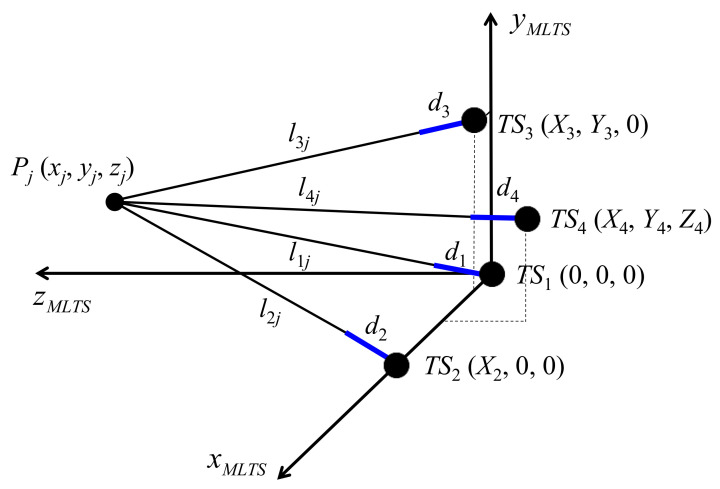
Schematic of the proposed self-calibration method for a multilateration tracking system using absolute distance measurement.

The schematic in Figure 2 depicts the proposed self-calibration method for an MLTS using ADM, in which the typical locations of four tracking stations are selected in the manner shown in Figure 1. In the IDM-based MLTS, the initial lengths vary depending on the position of the initial point. In contrast, the dead paths (*d*_1_, *d*_2_, *d*_3_, and *d*_4_) of an ADM-based MLTS, defined as the distance between the origin of ADM and the location of each tracking station, are fixed values. Thus, these parameters can be utilized continuously following calibration. Accordingly, the absolute distances from each tracking station to all target points can be attained.

Rather than simultaneously estimating all system parameters, the proposed self-calibration process for an ADM-based MLTS is divided into two steps: (1) estimation of dead paths and (2) estimation of tracking station locations. Four and six system parameters, respectively, are included in these two steps. Because a small number of system parameters are estimated in each step, the residual function complexity is reduced. This might enhance the performance of the self-calibration process. Because step (1) can be executed independently of step (2), the estimation of dead paths can be improved by using a high-precision reference positioning system, such as a coordinate measuring machine (CMM).

### 3.1. Step 1: Estimation of Dead Paths

#### 3.1.1. Estimation of Elevation Angle Offsets for Automated Initial Guess of System Parameters

The nonlinear least squares method is commonly used to estimate system parameters when a multilateration scheme is adopted [17,18,19,20,21]. Therefore, the initial guesses of tracking station locations should be sufficiently accurate to avoid convergence failure or a local minimum problem. Considering the spherical coordinate system at each tracking station, if comprehensive calibration of all geometric parameters (described in a previous study [22]) is skipped and only the elevation angle offset is roughly estimated with the dead path in advance, each tracking station can report the approximate 3D coordinates of target points with sufficient accuracy for an initial guess. The relationship of all tracking station locations can be determined by the best rigid transformation.

**Figure 3:**
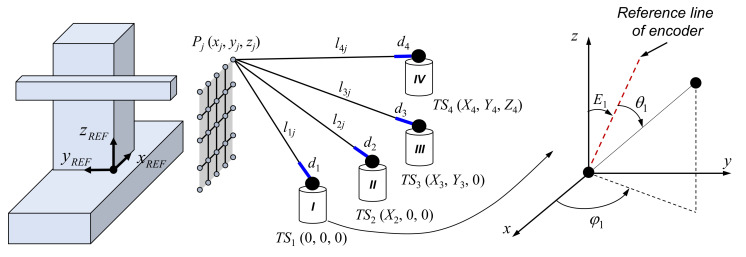
Schematic of estimating elevation angle offsets and initial guesses of dead paths.

The schematic in Figure 3 shows the estimation of elevation angle offsets and initial guesses of dead paths, whereby the four tracking stations are located in front of the grid of reference points provided by a high-precision positioning system. Under the assumption that the positioning system is sufficiently accurate, the distance between the reference points can be regarded as a fixed term (nominal distance provided by reference positioning system) in a least-squares adjustment. At tracking station 1, the elevation angle offset (*E*_1_) and initial guess (*d*_1*_ini*_) of dead path (*d*_1_) can be estimated by minimizing the residual function *R_ele_*_1_, as follows:

(3) $${\;R}_{ele1}\;=\;\overset{n-1}{\underset{j=1}{\sum }}\varepsilon_{ele1\_j}^{2}\;=\;\overset{n-1}{\underset{j=1}{\sum }}{\left\{{\left[{{(x}_{j}\;-{\;x}_{j+1})}^{2}{\;+\;(y}_{j}\;-{\;y}_{j+1}{)}^{2}{\;+\;(z}_{j}\;-{\;z}_{j+1}{)}^{2}\right]}^{1/2}-{\;l}_{ref\_j}\right\}}^{2}$$

where *n* is the number of reference points and *l_ref_j_* is the nominal distance between two reference points, *P_j_(x_j_, y_j_, z_j_)* and *P_j+_*_1_*(x_j+_*_1_*, y_j+_*_1_*, z_j+_*_1_*)*. These reference points are also provided in the local coordinates of tracking station 1, using the following formulas:

(4) $$x_{j}{\;=\;(l}_{1j}{\;+\;d}_{1\_ini}{)\;\times \;sin(\theta }_{1j}{\;+\;E}_{1}{)\;\times \;cos(\varphi }_{1j})$$

(5) $${\;y}_{j}{\;=\;(l}_{1j}{\;+\;d}_{1\_ini}{)\;\times \;sin(\theta }_{1j}{\;+\;E}_{1}{)\;\times \;sin(\varphi }_{1j})$$

(6) $$z_{j}{\;=\;(l}_{1j}{\;+\;d}_{1\_ini}{)\;\times \;cos(\theta }_{1j}{\;+\;E}_{1})\;$$

where *l*_1*j*_ is the measured absolute distance at tracking station 1, *θ*_1*j*_ is the measured elevation angle, and *φ*_1*j*_ is the measured azimuth angle. The elevation angle offsets (*E*_2_, *E*_3_, and *E*_4_) and the initial guesses (*d*_2*_ini*_, *d*_3*_ini*_, and *d*_4*_ini*_) of the dead paths of the remaining tracking stations are estimated in a similar manner.

#### 3.1.2. Estimation of Dead Paths

Because information concerning dead paths (*d*_1*_ini*_, *d*_2*_ini*_, *d*_3*_ini*_, and *d*_4*_ini*_) is obtained based on angle measurements, which are relatively inaccurate, these measurements are used only as the initial guesses for dead-path estimation. In contrast, only distance information is used for actual dead-path estimation. By using the same schematic shown in Figure 3, the residual function *R*_1_ for estimating the dead path of tracking station 1 is defined as the total sum of squares error *ε_d_*_1*_j*_, as follows:

(7) $${\;R}_{1}\;=\;\overset{n}{\underset{j=1}{\sum }}\varepsilon_{d1\_j}^{2}\;=\;\overset{n}{\underset{j=1}{\sum }}{\left\{{\left[{{(x}_{j}\;-{\;X}_{1})}^{2}{\;+\;(y}_{j}-{\;Y}_{1}{)}^{2}{\;+\;(z}_{\;j}\;-{\;Z}_{1}{)}^{2}\right]}^{1/2}-{\;(d}_{1}{\;+\;l}_{1j})\right\}}^{2}$$

where *x_j_*, *y_j_*, and *z_j_* are the coordinates of the reference target point, while *X*_1_, *Y*_1_, *Z*_1_, and *d*_1_ are the location and dead path of tracking station 1, respectively. Here, *x_j_*, *y_j_*, *z_j_*, and *X*_1_, *Y*_1_, *Z*_1_ are the coordinates defined by the reference positioning system.

**Figure 4:**
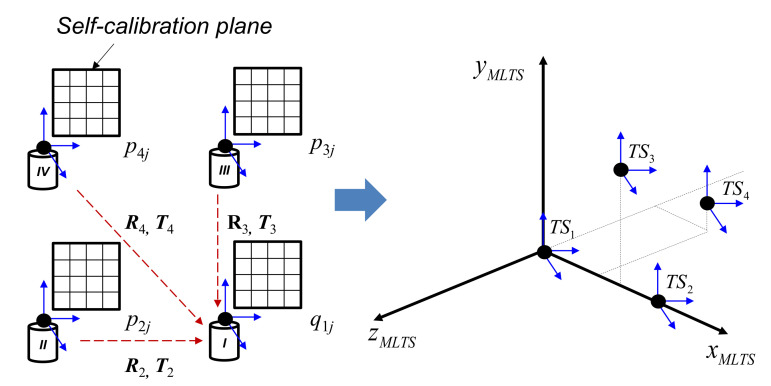
Transformation from local coordinates to global multilateration tracking system (MLTS) coordinates.

Denoting ***W***_1_ as the vector of *X*_1_, *Y*_1_, *Z*_1_, and *d*_1_, ***W***_1_ is estimated by an iterative solution based on minimization of *R*_1_, as follows:

(8) $$W_{1}^{k+1}{\;=W}_{1}^{k}\;+\;\Delta_{1}^{k}$$

where iteration step *k =* 1, 2*… m*, ***W***_1_*^k=^*^1^ is the initial guess of ***W*_1_**, and **Δ**_1_ is the vector of a correction term. Notably, *X*_1_, *Y*_1_, and *Z*_1_ of ***W***_1_*^k=^*^1^ can be determined using a predefined layout and technical drawings, but they are determined more conveniently and accurately by using the estimated *E*_1_ and *d*_1*_ini*_. The coordinates of target points expressed in the reference positioning system can also be expressed in the local coordinate system of tracking station 1. The initial guesses of *X*_1_, *Y*_1_, and *Z*_1_ can be obtained through coordinate matching between the two coordinate systems. A detailed description of the process has been omitted because it is similar to the method described in Section 3.2 and Figure 4.

Denoting ***L***_1_ as a vector of the residual error *ε_d_*_1__*_j_* at current guess value of ***W***_1_, **Δ**_1_ is determined by taking the derivative of *R*_1_ with respect to **Δ**_1_, as follows:

(9) $$\frac{\partial R_{1}}{\partial \Delta_{1}}\;=-J^{T}L_{1}{\;+\;J}^{T}J\Delta_{1}$$

where ***J*** is a Jacobian matrix. Setting $\frac{\partial R_{1}}{\partial \Delta_{1}}\;$= 0, the normal equation for determining **Δ_1_** in each iteration step is obtained as follows:

(10) $$J^{T}J\Delta_{1}{\;=\;J}^{T}L_{1}$$

To guarantee numerical stability, Equation (10) is solved using singular value decomposition or QR decomposition. The iterative process is stopped when the difference between two consecutive values of ***W***_1_ is smaller than a convergence criterion (a typical value is approximately 10^−8^). The dead paths of the remaining tracking stations are estimated in a similar manner.

### 3.2. Step 2: On-Site Calibration of the Tracking Station Locations

After estimation of dead paths and elevation angle offsets using the high-precision positioning system, four tracking stations can be installed for real 3D coordinate measurements based on the MLTS principle. Because each tracking station reports the approximate coordinates of the target points in its local coordinate system, a transformation step shown in Figure 4 is performed to obtain the initial guesses of the tracking station locations in the global MLTS coordinate system.

In detail, the relationships among all tracking stations (rotation matrix ***R*** and translation matrix ***T***) are identified by applying the best-fitting rigid transformation from the local coordinates (***p****_j_*) of tracking stations 2–4 to the local coordinates (***q****_j_*) of tracking station 1. The principle is based on minimization of the function *R_trans_*, as follows:

(11) $${\;R}_{trans}\;=\;\overset{n}{\underset{j=1}{\sum }}\|\left({Rp}_{j}\;+\;T\right){-\;q}_{j}\|{}^{2}$$

Subsequently, the locations of tracking stations 2–4 are found in the local coordinate system of tracking station 1. This essential information is then transferred into the global Cartesian coordinates of the MLTS, in which tracking station 1 is regarded as the origin, tracking station 2 is on the x-axis, tracking station 3 is on the xy-plane, and tracking station 4 is out of the xy-plane. Following this transfer into the global MLTS coordinates to obtain the initial guesses of the tracking station locations, the vector of the tracking station locations is estimated by minimization of the residual function *R_on_site_*, defined as:

(12) $${\;R}_{on\_siee}\;=\;\overset{n}{\underset{j=1}{\sum }}\varepsilon_{j}^{2}\;=\;\overset{n}{\underset{j=1}{\sum }}{\left\{{\left[{{(x}_{j}\;{-\;X}_{4})}^{2}{\;+\;(y}_{j}\;{-\;Y}_{4}{)}^{2}{\;+\;(z}_{j}\;{-\;Z}_{4}{)}^{2}\right]}^{1/2}{-\;(d}_{4}{\;+\;l}_{4j})\right\}}^{2}$$

where, *l*_1*j*_, *l*_2*j*_, *l*_3*j*_, and *l*_4*j*_ are measured absolute distances and *d*_1_, *d*_2_, *d*_3_, and *d*_4_ are the dead paths of tracking stations 1, 2, 3, and 4, respectively. The *x_j_*, *y_j_*, *z_j_* coordinates are given by the following trilateration formula:

(13) $$x_{j}\;=\;\;\frac{{{(d}_{1}{\;+\;l}_{1j})}^{2}\;{-\;(d}_{2}{\;+\;l}_{2j}{)}^{2}{\;+\;X}_{2}^{2}}{{2X}_{2}}$$

(14) $${\;y}_{j}\;=\;\frac{{{(d}_{1}{\;+\;l}_{1j})}^{2}\;{-\;(d}_{3}{\;+\;l}_{3j}{)}^{2}{\;+\;X}_{3}^{2}{\;+\;Y}_{3}^{2}\;{-\;2X}_{3}{.x}_{j}}{{2Y}_{3}}$$

(15) $$z_{j}\;=\;{\left({{(d}_{1}{\;+\;l}_{1j})}^{2}\;{-\;x}_{j}^{2}\;{-\;y}_{j}^{2}\right)}^{1/2}$$

**Figure 5:**
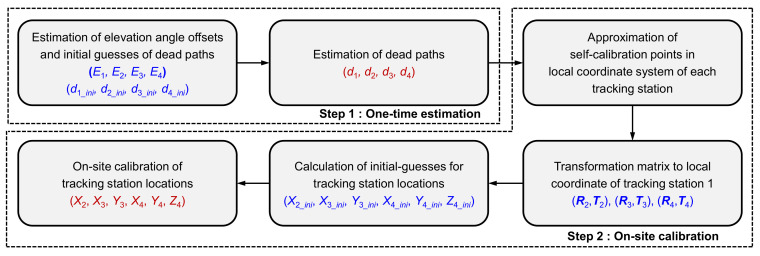
Flow chart of the proposed self-calibration method for an absolute distance measurement (ADM)-based MLTS.

The flow chart shown in Figure 5 summarizes the proposed self-calibration method for estimation of system parameters, including dead paths and tracking station locations.

## 4. Experimental Results

### 4.1. Experiment Setup

**Figure 6:**
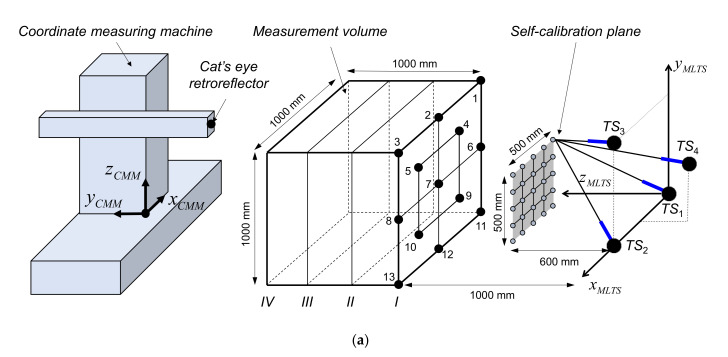

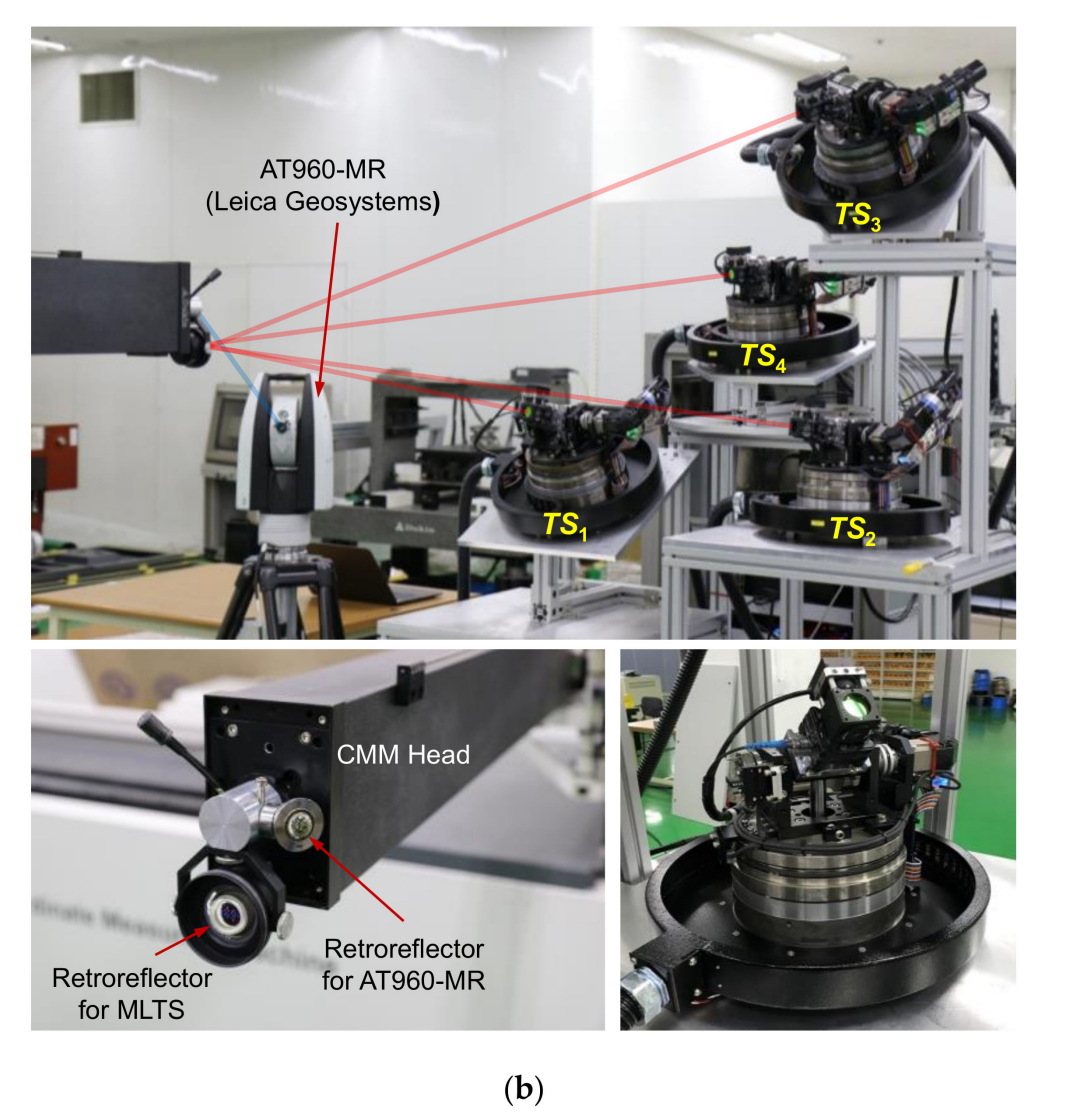
Experimental setup for self-calibration and 3D coordinate measurement: (**a**) Schematic of experimental setup; (**b**) Photograph of experimental setup.

Figure 6a shows the experimental setup for self-calibration and 3D coordinate measurement. The layout was considered first [11,13,17]. Then, based on actual experimental conditions with respect to the acceptance angle of the retroreflector and the rotation limitations of each tracking station, the nominal layout was selected as follows (all in mm): *TS*_1_(0, 0, 0), *TS*_2_(700, 0, 0), *TS*_3_(700, 700, 0), and *TS*_4_(350, 350, −350).

The 25 target points chosen for self-calibration were uniformly distributed in a 500 mm × 500 mm square area that was approximately 600 mm from the xy-plane of the MLTS. The 52 points selected for actual 3D coordinate measurement were equally distributed in four parallel planes in a 1000 mm × 1000 mm × 1000 mm volume, which was located approximately 1000 mm from the xy-plane of the MLTS. To provide target position measurement for self-calibration and 3D coordinate measurement, a cat’s eye retroreflector was installed at the CMM (DUKIN, GIANT-251515). To further verify the efficiency and feasibility of the proposed method, a commercial laser tracker from Leica Geosystems (AT960-MR, Heerbrugg, Switzerland), for which the accuracy was ± 15 μm + 6 μm/m, was used to provide comparable 3D coordinate measurement results. The experiment for the self-calibration process was designed first. A 3D coordinate measurement based on the estimated system parameters was then conducted and the measured 3D coordinates were compared with the coordinates determined by AT960MR. The experiment was carried out in a laboratory environment where the temperature was controlled to 20 ± 1 °C.

As shown in Figure 6b, the tracking station carries the optic module for ADM. Basically, the tracking station is a combination of precision ball bearings and motors that can create two-way rotational motion. However, using a reference ball, the optical axis and two rotation axes were carefully aligned to match each other to reduce the measurement error caused by geometric misalignment [9,22]. The resolutions of the encoders used for azimuth and elevation axis were approximately 0.21 arcsec and 0.79 arcsec, respectively.

**Figure 7:**
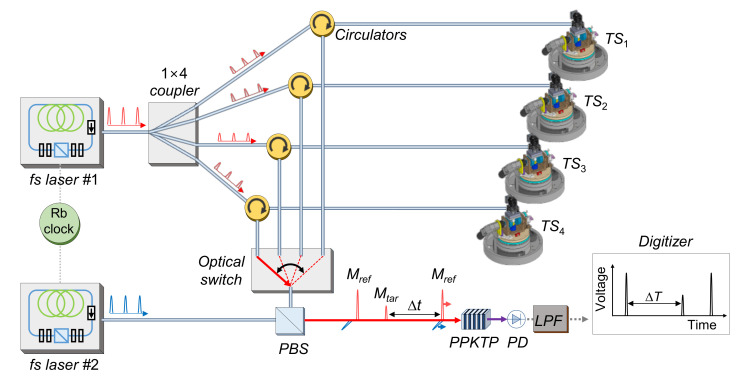
Configuration of absolute distance measurement for the MLTS (fs: femtosecond).

Figure 7 shows the configuration of the ADM for the MLTS. The ADM module was designed to measure the absolute distance between each tracking station and the retroreflector, in which the dead path was excluded. In the ADM, a time-of-flight method involving asynchronous optical sampling was applied to measure long distance with high precision [6,23,24,25].

For asynchronous optical sampling, two femtosecond lasers with slightly different repetition rates were used. Femtosecond laser #1 generated femtosecond laser pulses with a repetition rate of approximately 100 MHz (*f_r_*). The power of the generated laser pulses was divided into four directions by a 1 × 4 coupler and then delivered to each tracking station through an optical fiber. Some of the laser pulses arriving at the tracking stations were reflected back from the reference plane (*M_ref_*). The remaining transmitted pulses were reflected by the retroreflector and returned back through the optical fiber (*M_tar_*). The reflected laser pulses (*M_ref_* and *M_tar_*) were coupled with laser pulses from femtosecond laser #2 in a polarization state perpendicular to each other in the polarizing beam splitter (PBS), and were incident on the nonlinear optical crystal (PPKTP). At this point, due to the difference in the repetition rates (Δ*f_r_*, approximately 2 kHz) of the two femtosecond lasers, the laser pulses of femtosecond laser #1 were scanned and sampled by the laser pulses of femtosecond laser #2. The absolute distance *l* from reference plane to the retroreflector was determined as follows:

(16) $$l\;=\;c\;\frac{\Delta t}{2}\;=\;c\;\frac{\Delta T}{2}\;(\frac{\Delta f_{r}}{f_{r}})$$

where *c* is the speed of light in the air, Δ*t* is the delay of the laser pulse, and Δ*T* is the delay of the sampled radiofrequency signal. The absolute distances from each tracking station were measured sequentially while switching the high-speed optical switch.

### 4.2. Simulation and Experimental Verification of the Proposed Self-Calibration Method

**Figure 8:**
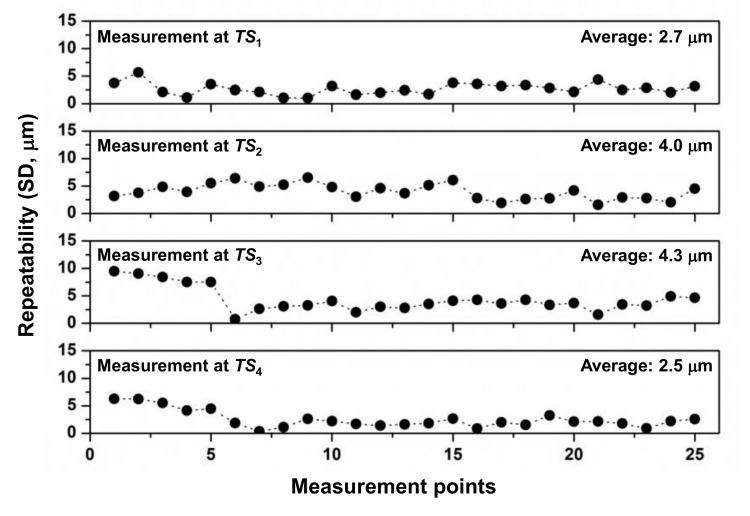
Repeatability of absolute distance measurement for 25 target points used for self-calibration (SD: standard deviation).

With the experimental setup shown in Figure 6, the repeatability of the ADM, which is represented by standard deviation, was first analyzed for five measurements. One measurement value of the absolute distance was the average of 100 measurement samples. Figure 8 shows the repeatability for 25 target points used for self-calibration.

In the previous studies that measured absolute distances in a laboratory condition, the distances were measured with repeatability of 0.21 µm [6] and 0.22 µm [25] using an average of 100 samples. However, in this experiment, the distance was measured with repeatability of several micrometers. The presumed main factor in performance deterioration was due to distortion of the laser pulse caused by mixing a polarization-maintaining (PM) fiber and a non-polarization-maintaining (non-PM) fiber. This introduced unwanted polarization components in the PM fiber and caused signal distortion by polarization mode dispersion. Another presumed contributing factor was the poor stiffness of the MLTS structure, which is composed of aluminum profile. These aspects will be improved in future experiments.

#### 4.2.1. Simulation of the Proposed Self-Calibration Method

To verify the efficiency and feasibility of the proposed method, the MLTS was simulated by using the conventional (IDM-based) and proposed (ADM-based) self-calibration methods. To match the experimental and simulation conditions, a layout identical to that used in Figure 6a was implemented during simulation. For simulation of the proposed method, the true value of the dead path was defined as −40 mm. Following the comment of a previous study [14] that the initial point should not be too close from the self-calibration plane, point No. 7 in the measurement volume was selected as an initial point during simulation of the conventional method.

To avoid an excessively complex simulation, the proposed calculation of initial guesses was excluded from the simulation. Instead, initial guesses that differed by ±5 mm from the true values were randomly generated and used in the simulation. The measurement errors were simulated under conditions identical to those of the experimental system, such that the average standard deviation (σ) of the ADM was approximately 3.4 µm. The maximum error of the target points provided by the CMM was assumed to be 5 µm in the x-, y-, and z-directions. In the simulation, the errors of the ADM (or IDM) and position errors of the target points were randomly generated obeying a Gaussian distribution within [−10 µm, 10 µm] and [−5 µm, 5 µm], respectively. Numerical simulations were performed using commercial MATLAB software and were performed by following the relevant steps of the conventional and proposed methods. Among the codes, the function normrnd (µ, σ) was used to generate random errors with a Gaussian distribution (mean µ, standard deviation σ). To solve the Equation (10) efficiently, the function pinv (J^T^J) was used to perform pseudo inverse computation based on singular value decomposition.

**Figure 9:**
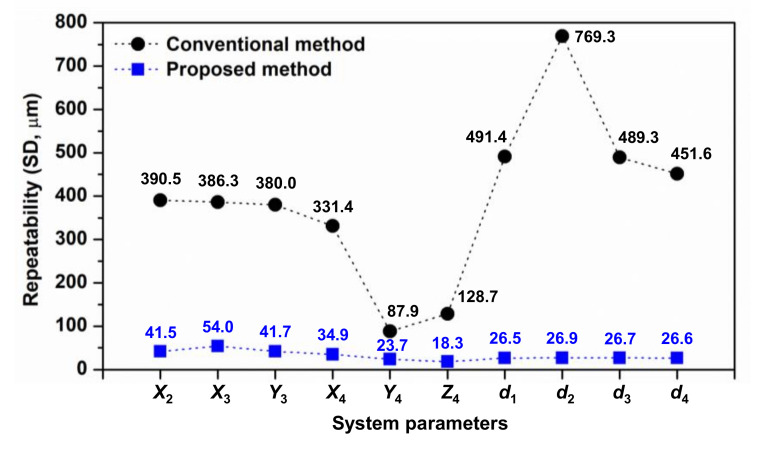
Estimation repeatability of system parameters using proposed and conventional self-calibration methods (simulation).

The self-calibration process was simulated 10,000 times. Because errors with a Gaussian distribution were used in the simulation, the average of the system parameters obtained in each simulation approximated the true value. Therefore, only the repeatability of the estimated system parameters by the two methods was compared. These values are shown in Figure 9.

Simulation showed that the proposed self-calibration method provided estimated system parameters that were more stable than those estimated by the conventional method. In particular, the estimation repeatability of the system parameters obtained by the conventional method ranged from 87.9 to 769.3 μm, whereas that of those obtained by the proposed method ranged from 18.3 to 54.0 μm. These values were approximately 3.7-fold better at the smallest difference (i.e., estimation of *Y*_4_) and 28.6-fold better at the largest difference (i.e., estimation of *d*_2_). These findings show that the proposed two-step approach was very effective for improving self-calibration performance by reducing the number of system parameters in each step.

#### 4.2.2. Experimental Results of the Proposed Self-Calibration Method

The self-calibration experiment was carried out three times in the order shown in Figure 5. First, the elevation angle offsets (*E*_1_, *E*_2_, *E*_3_, and *E*_4_) and initial guesses of the dead paths (*d*_1*_ini*_, *d*_2*_ini*_, *d*_3*_ini*_, and *d*_4*_ini*_) were estimated using 25 points on the self-calibration plane shown in Figure 6a. Then, dead paths (*d*_1_, *d*_2_, *d*_3_, and *d*_4_) were estimated using identical ADM data. Next, without the aid of external devices, the initial guesses for the tracking station locations were obtained in accordance with the method described in Section 3.2. Finally, the system parameters of the tracking station locations (*X*_2_, *X*_3_, *Y*_3_, *X*_4_, *Y*_4_, and *Z*_4_) were estimated by minimizing the residual function shown in Equation (12). To compare estimation results, system parameters were also estimated by the conventional method. As in the simulation, point No. 7 in the measurement volume was selected as an initial point to convert ADM data to IDM data.

Table 1 shows the system parameters estimated by the proposed and conventional methods. Combining the results of three experiments, the initial guesses showed very small differences of less than 0.5 mm from the final estimated values, which was sufficient to avoid convergence failure or local minimum problems. This demonstrates that the proposed method for acquisition of initial guesses was very effective. For reference, initial guesses obtained by the proposed self-calibration method were also used for the conventional self-calibration process. The system parameters obtained by the two methods showed the following maximum differences: 0.918 mm in Experiment 1, 0.429 mm in Experiment 2, and 0.324 mm in Experiment 3.

**Figure 10:**
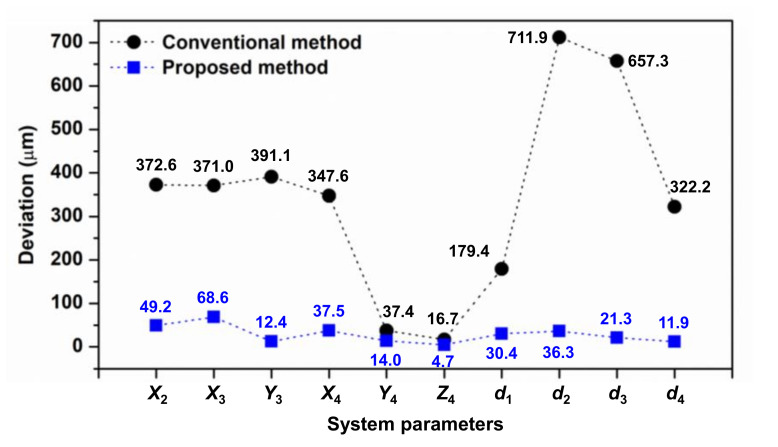
Deviation of the estimated system parameters in three experiments.

The deviations of the estimated system parameters listed at the bottom of Table 1 are visualized and shown in Figure 10. Compared with the simulation results shown in Figure 9, the two results demonstrate a very similar trend. In the three experiments, the deviation of the estimated system parameter obtained by the conventional method ranged from 16.7 to 711.9 μm, whereas the deviation obtained by the proposed method ranged from 4.7 to 68.6 μm. These values are approximately 2.7-fold better at the smallest difference (i.e., estimation of *Y*_4_) and 31.6-fold better at the largest difference (i.e., estimation of *Y*_3_).

### 4.3. Experimental Verification of 3D Coordinate Measurement Performance

The experiment of 3D coordinate measurement was carried out three times by using the corresponding system parameters shown in Table 1. The 3D coordinates of 52 target points in the measurement volume (Figure 6a) were calculated by a trilateration formula (Equations (13)–(15)) using the absolute distances measured at each tracking station. Simultaneously, the 3D coordinates of the target points were measured by a commercial laser tracker (AT960-MR, Heerbrugg, Switzerland). Because the coordinate systems differed between the MLTS and the AT960-MR, the 3D coordinates measured by the two measuring devices were transformed to a CMM coordinate system and then compared.

**Figure 11:**
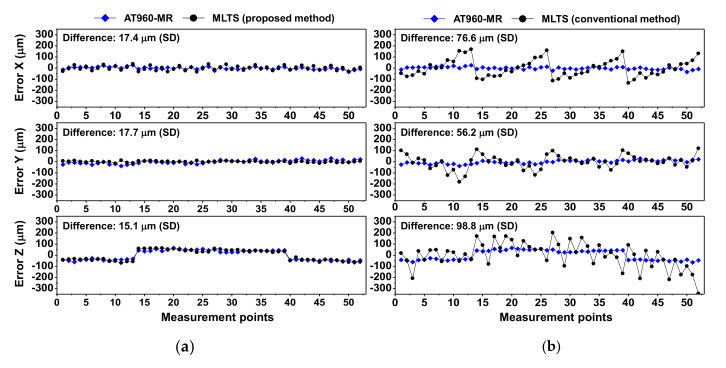
Comparison of coordinate measuring machine (CMM) errors measured by AT960-MR and MLTS in Experiment 1: (**a**) Proposed self-calibration method; (**b**) Conventional self-calibration method.

Figure 11a shows CMM errors in the x-, y- and z-directions in Experiment 1, as measured by the AT960-MR and MLTS with the proposed self-calibration method. Based on the standard deviation, the differences between measurement results for 52 points were 17.4 μm in the x-direction, 17.7 μm in the y-direction, and 15.1 μm in the z-direction. Figure 11b shows CMM errors, as measured by the AT960-MR and MLTS using the conventional method. The respective differences were 76.6 μm, 56.2 μm, and 98.8 μm. These findings indicate that the CMM errors measured by the proposed method were in good agreement with the errors measured by the AT960-MR.

**Figure 12:**
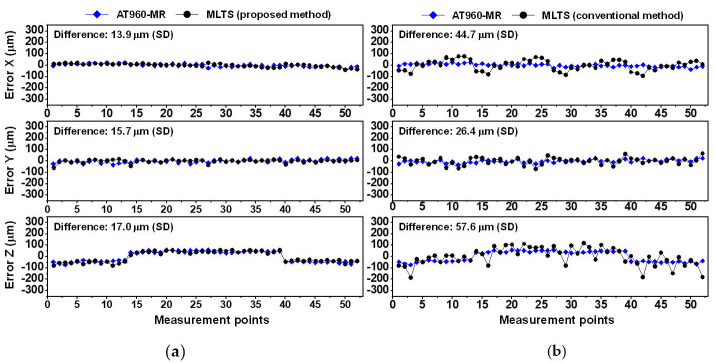
Comparisons of CMM errors measured by AT960-MR and MLTS in Experiment 2: (**a**) Proposed self-calibration method; (**b**) Conventional self-calibration method.

**Figure 13:**
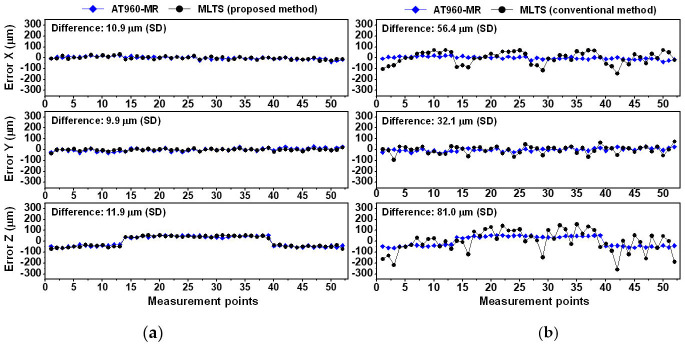
Comparison of CMM errors measured by AT960-MR and MLTS in Experiment 3: (**a**) Proposed self-calibration method; (**b**) Conventional self-calibration method.

Figure 12 and Figure 13 show comparisons of the CMM errors obtained in Experiments 2 and 3. The overall trends were similar to the trend in Experiment 1. The CMM errors measured by the conventional method were not repetitive and differed considerably from the errors measured by the AT960-MR. These results confirmed that the system parameters estimated by the proposed method were more accurate than those estimated by the conventional method.

## 5. Conclusions

Herein, we presented a custom-designed self-calibration method for estimating dead paths and tracking station locations in an ADM-based MLTS. The advantage of ADM is that the dead path comprises a fixed value, which enables use of the proposed two-step self-calibration approach. Therefore, a small number of system parameters are estimated in each step, which reduces the complexity of the original residual function. In addition, dead path estimation accuracy is further enhanced by the use of a high-precision positioning system, which is available in this separate calibration step. Based on the rough estimation of elevation angle offset in step (1), a method is presented to calculate the initial guesses of the system parameters automatically. Using the appropriate mathematical procedure and relevant solution of initial guesses significantly improves the estimation performance of the system parameters.

The effectiveness of the proposed self-calibration method was demonstrated by simulation and experimental approaches. Compared with the conventional method, the proposed method showed improved repeatability during estimation of system parameters. In three self-calibration experiments, the maximum deviation of the estimated system parameters obtained by the proposed method was 68.6 µm, while the maximum deviation obtained by the conventional method was 711.9 µm. Furthermore, by applying the proposed calculation process of initial guess, the initial guesses differed by less than 0.5 mm from the final estimates, which was sufficient to avoid convergence failure or local minimum problems. Importantly, validation of 3D coordinate measurements in a 1000 mm× 1000 mm × 1000 mm volume showed that measurement results based on the proposed method were consistent with those obtained using a commercial laser tracker. Differences between the two measurement results were 10.9–17.4 μm in the x-direction, 9.9–17.7 μm in the y-direction, and 11.9–17.0 μm in the z-direction. Differences between the two measurements based on the conventional method, and using a commercial laser tracker, were 44.7–76.6 μm in the x-direction, 26.4–56.2 μm in the y-direction, and 57.6–98.8 μm in the z-direction.

These results show that the proposed ADM-based MLTS can provide much more precise 3D coordinate measurements than the IDM-based MLTS, provided the performance of ADM and IDM is the same. Although the current performance of 3D coordinate measurement is comparable to that of a commercial laser tracker, the repeatability of the self-made ADM system integrated with MLTS is only 3.4 μm under experimental condition. It shows the performance gap with currently available IDM-based MLTS. For example, measuring uncertainty of LASERTRACER by ETALON is 0.2 μm + 0.3 μm/m for spatial displacement. In the near future, we expect that the repeatability of ADM could be less than 0.5 μm at 50 ms sampling time, and this improvement could bring a proportional performance enhancement of 3D coordinate measurements. ADM-based MLTS has obvious advantages in large-scale 3D coordinate measurements compared to commercial laser trackers, but the increased system complexity and cost due to the need for four tracking stations are limiting factors for a wide range of applications. Low-cost multi-channel ADM and miniaturized tracking stations are key to overcoming these limitations.

## Figures and Tables

**Table 1:** Estimation results of system parameters by using proposed and conventional self-calibration methods (unit: mm).

		X2	X3	Y3	X4	Y4	Z4	d1	d2	d3	d4
Exp. 1	Initial Guess (a)	700.766	697.037	697.272	346.868	348.121	−348.487	−42.497	−42.952	−42.725	−42.892
	Proposed (b)	701.061	696.813	697.628	346.761	348.218	−348.735	−42.266	−42.464	−42.693	−42.531
	Conventional (c)	701.218	697.466	697.984	347.088	348.346	−348.842	−42.168	−42.110	−41.775	−42.062
	(a)–(b)	−0.295	0.224	−0.356	0.108	−0.097	0.249	−0.230	−0.488	−0.032	−0.361
	(c)–(b)	0.157	0.654	0.356	0.327	0.128	−0.107	0.099	0.353	0.918	0.470
Exp. 2	Initial Guess (a)	700.983	697.040	697.312	346.884	348.107	−348.464	−42.494	−42.743	−42.677	−42.895
	Proposed (b)	701.012	696.744	697.616	346.723	348.229	−348.731	−42.292	−42.500	−42.708	−42.540
	Conventional (c)	701.006	697.112	697.726	346.852	348.331	−348.825	−42.155	−42.454	−42.279	−42.228
	(a)–(b)	−0.028	0.296	−0.304	0.161	−0.122	0.267	−0.202	−0.243	0.031	−0.355
	(c)–(b)	−0.006	0.368	0.110	0.129	0.102	−0.095	0.137	0.046	0.429	0.312
Exp. 3	Initial Guess (a)	700.807	697.114	697.300	346.877	348.195	−348.457	−42.493	−42.917	−42.599	−42.878
	Proposed (b)	701.031	696.806	697.620	346.751	348.232	−348.735	−42.297	−42.499	−42.687	−42.543
	Conventional (c)	700.845	697.095	697.593	346.740	348.368	−348.835	−42.335	−42.822	−42.433	−42.384
	(a)–(b)	−0.224	0.308	−0.319	0.126	−0.037	0.278	−0.197	−0.418	0.088	−0.335
	(c)–(b)	−0.186	0.290	−0.026	−0.011	0.137	−0.100	−0.038	−0.324	0.254	0.159
Deviation	Proposed (d)	0.049	0.069	0.012	0.038	0.014	0.005	0.030	0.036	0.021	0.012
	Conventional (e)	0.373	0.371	0.391	0.348	0.037	0.017	0.179	0.712	0.657	0.322
	Ratio ((e)/(d))	7.6	5.4	31.6	9.3	2.7	3.6	5.9	19.6	30.8	27.0
